# Supplementary material for: Shock propagation channels behind the global economic contagion network. The role of economic sectors and the direction of trade
Source: PLoS One. 2021 Oct 20;16(10):e0258309. doi: 10.1371/journal.pone.0258309 (PMC8528308; doi:10.1371/journal.pone.0258309)
Supplement: S1 Appendix — This document contains the detailed conditional logit regression results (tables) for every model version that was estimated. The regression results are ordered according to Table 3. (PDF) [file pone.0258309.s001.pdf]

Model – 1

```
. clogit gdppc popi popj gdpcapi gdpcapj op, or group(countrypair) robust
note: multiple positive outcomes within groups encountered.
note: 808 groups (33936 obs) dropped because of all positive or
      all negative outcomes.

Iteration 0:   log pseudolikelihood = -15443.265
Iteration 1:   log pseudolikelihood = -14980.261
Iteration 2:   log pseudolikelihood = -14952.965
Iteration 3:   log pseudolikelihood = -14952.434
Iteration 4:   log pseudolikelihood = -14952.433

Conditional (fixed-effects) logistic regression   Number of obs   =      38388
                                                  Wald chi2(5)    =      56.73
                                                  Prob > chi2     =     0.0000
Log pseudolikelihood = -14952.433                 Pseudo R2       =     0.0308

                            (Std. Err. adjusted for clustering on countrypair)
```

gdppc	Odds Ratio	Robust Std. Err.	z	P>|z|	[95% Conf.	Interval]
popi	.9848761	.0056498	-2.66	0.008	.9738647	.996012
popj	1.06074	.0107065	5.84	0.000	1.039962	1.081934
gdpcapi	.8874647	.0226883	-4.67	0.000	.8440922	.9330659
gdpcapj	1.152534	.0325587	5.03	0.000	1.090454	1.218147
op	1.010309	.0052051	1.99	0.047	1.000159	1.020563

Variables:

gdppc: dependent binary variable, if the value is 1, the cyclical components of country *i* granger causes the cyclical components of country *j*, so the shock spreads
gdpcapi: per capita GDP of country *i*
gdpcapj: per capita GDP of country *j*
popi: population in country *i*
popj: population in country *j*
op: the average trade volume between country *i* and country *j* divided by the GDP of country *j*

Model – 2A

```
Conditional (fixed-effects) logistic regression   Number of obs   =      38388
                                                  Wald chi2(6)    =      57.42
                                                  Prob > chi2     =     0.0000
Log pseudolikelihood = -14946.345                 Pseudo R2       =     0.0312

                            (Std. Err. adjusted for clustering on countrypair)
```

gdppc	Odds Ratio	Robust Std. Err.	z	P>|z|	[95% Conf.	Interval]
popi	.9852538	.0056001	-2.61	0.009	.9743387	.9962911
popj	1.060491	.0106602	5.84	0.000	1.039801	1.081592
gdpcapi	.8876793	.0227075	-4.66	0.000	.8442706	.9333198
gdpcapj	1.15319	.0327404	5.02	0.000	1.090773	1.219179
up	.9990693	.0071911	-0.13	0.897	.9850739	1.013264
down	1.010078	.0086875	1.17	0.244	.9931932	1.027249

Variables:

up: average trade volume from country *j* to country *i* divided by the GDP of country *j*
down: average trade volume from country *i* to country *j* divided by the GDP of country *j*

Model – 2B

```
Conditional (fixed-effects) logistic regression   Number of obs   =      38388
                                                   Wald chi2(5)    =      55.14
                                                   Prob > chi2     =     0.0000
Log pseudolikelihood = -14966.836                  Pseudo R2       =     0.0298

                              (Std. Err. adjusted for clustering on countrypair)
```

gdppc	Odds Ratio	Robust Std. Err.	z	P>|z|	[95% Conf.	Interval]
popi	.9851711	.0056486	-2.61	0.009	.9741621	.9963046
popj	1.060582	.010671	5.85	0.000	1.039872	1.081704
gdpcapi	.8907022	.0227005	-4.54	0.000	.847303	.9363244
gdpcapj	1.147854	.0321268	4.93	0.000	1.086582	1.21258
up	1.006458	.0051588	1.26	0.209	.9963979	1.01662

```
Conditional (fixed-effects) logistic regression   Number of obs   =      38388
                                                   Wald chi2(5)    =      57.40
                                                   Prob > chi2     =     0.0000
Log pseudolikelihood = -14946.465                  Pseudo R2       =     0.0312

                              (Std. Err. adjusted for clustering on countrypair)
```

gdppc	Odds Ratio	Robust Std. Err.	z	P>|z|	[95% Conf.	Interval]
popi	.9851722	.0055745	-2.64	0.008	.9743068	.9961589
popj	1.060542	.0106651	5.85	0.000	1.039844	1.081653
gdpcapi	.8874509	.0225769	-4.69	0.000	.844286	.9328226
gdpcapj	1.153347	.0325802	5.05	0.000	1.091227	1.219004
down	1.009577	.0062053	1.55	0.121	.9974877	1.021813

Model – 3A

```
Conditional (fixed-effects) logistic regression   Number of obs   =      34152
                                                   Wald chi2(14)   =      94.89
                                                   Prob > chi2     =     0.0000
Log pseudolikelihood = -13043.069                  Pseudo R2       =     0.0479

                              (Std. Err. adjusted for clustering on countrypair)
```

gdppc	Odds Ratio	Robust Std. Err.	z	P>|z|	[95% Conf.	Interval]
popi	.9857961	.0056572	-2.49	0.013	.9747703	.9969466
popj	1.057231	.0095421	6.17	0.000	1.038693	1.076099
gdpcapi	.8703738	.02564	-4.71	0.000	.8215436	.9221063
gdpcapj	1.153791	.0371739	4.44	0.000	1.083184	1.229
op0	1.141174	.0663777	2.27	0.023	1.018218	1.278978
op1	.8490814	.1564053	-0.89	0.374	.5917707	1.218274
op2	.8805054	.0790302	-1.42	0.156	.7384685	1.049862
op3	.9497904	.0300455	-1.63	0.103	.8926907	1.010542
op4	2.853801	1.385118	2.16	0.031	1.102263	7.388595
op5	.8841636	.0356957	-3.05	0.002	.8168977	.9569684
op6	.9611029	.0255927	-1.49	0.136	.9122286	1.012596
op7	1.05266	.0190985	2.83	0.005	1.015886	1.090766
op8	1.185209	.0792218	2.54	0.011	1.039678	1.351111
op9	.9737451	.0328815	-0.79	0.431	.911385	1.040372

Variables:

op0: the average trade volume of *'Food and live animals'* industry between country *i* and country *j* divided by the GDP of country *j*

op1: the average trade volume of *'Beverages and tobacco'* industry between country *i* and country *j* divided by the GDP of country *j*

op2: the average trade volume of *'Crude materials, inedible, except fuels'* industry between country *i* and country *j* divided by the GDP of country *j*

op3: the average trade volume of *'Mineral fuels, lubricants and related materials'* industry between country *i* and country *j* divided by the GDP of country *j*
op4: the average trade volume of *'Animal and vegetable oils, fats and waxes'* industry between country *i* and country *j* divided by the GDP of country *j*
op5: the average trade volume of *'Chemicals and related products, n.e.s.'* industry between country *i* and country *j* divided by the GDP of country *j*
op6: the average trade volume of *'Manufactured goods classified chiefly by material'* industry between country *i* and country *j* divided by the GDP of country *j*
op7: the average trade volume of *'Machinery and transport equipment'* industry between country *i* and country *j* divided by the GDP of country *j*
op8: the average trade volume of *'Miscellaneous manufactured articles'* industry between country *i* and country *j* divided by the GDP of country *j*
op9: the average trade volume of *'Commodities and transactions not classified elsewhere in the SITC'* industry between country *i* and country *j* divided by the GDP of country *j*

Model – 3B

```
Conditional (fixed-effects) logistic regression   Number of obs   =      38346
                                                  Wald chi2(5)    =      60.46
                                                  Prob > chi2     =     0.0000
Log pseudolikelihood = -14910.294                 Pseudo R2       =     0.0318

                                (Std. Err. adjusted for clustering on countrypair)
```

gdppc	Odds Ratio	Robust Std. Err.	z	P>|z|	[95% Conf.	Interval]
popi	.9858545	.0055439	-2.53	0.011	.9750483	.9967805
popj	1.060808	.0106896	5.86	0.000	1.040062	1.081967
gdpcapi	.8886576	.0224184	-4.68	0.000	.8457869	.9337013
gdpcapj	1.143783	.0316732	4.85	0.000	1.083359	1.207577
op0	1.111143	.0438052	2.67	0.008	1.02852	1.200404

.

```
Conditional (fixed-effects) logistic regression   Number of obs   =      38041
                                                  Wald chi2(5)    =      56.31
                                                  Prob > chi2     =     0.0000
Log pseudolikelihood = -14818.455                 Pseudo R2       =     0.0296

                                (Std. Err. adjusted for clustering on countrypair)
```

gdppc	Odds Ratio	Robust Std. Err.	z	P>|z|	[95% Conf.	Interval]
popi	.9857111	.0055721	-2.55	0.011	.9748502	.996693
popj	1.058889	.0101971	5.94	0.000	1.039091	1.079065
gdpcapi	.8889666	.0226059	-4.63	0.000	.8457459	.9343961
gdpcapj	1.149167	.0325611	4.91	0.000	1.087088	1.214791
op1	1.188069	.1342605	1.52	0.127	.9520276	1.482633

```
Conditional (fixed-effects) logistic regression   Number of obs   =      38346
                                                  Wald chi2(5)    =      55.46
                                                  Prob > chi2     =     0.0000
Log pseudolikelihood = -14966.059                 Pseudo R2       =     0.0293

                                (Std. Err. adjusted for clustering on countrypair)
```

gdppc	Odds Ratio	Robust Std. Err.	z	P>|z|	[95% Conf.	Interval]
popi	.986206	.0056021	-2.45	0.014	.9752869	.9972473
popj	1.059989	.0105344	5.86	0.000	1.039542	1.080838
gdpcapi	.8952269	.022329	-4.44	0.000	.8525154	.9400783
gdpcapj	1.143124	.0316963	4.82	0.000	1.082658	1.206967
op2	.9705471	.0785137	-0.37	0.712	.8282426	1.137302

Conditional (fixed-effects) logistic regression   Number of obs = 36610
Wald chi2(5) = 57.00
Prob > chi2 = 0.0000
Log pseudolikelihood = -14253.377   Pseudo R2 = 0.0291

(Std. Err. adjusted for clustering on countrypair)

gdppc	Odds Ratio	Robust Std. Err.	z	P>|z|	[95% Conf.	Interval]
popi	.9873803	.0055938	-2.24	0.025	.9764772	.9984051
popj	1.056118	.0095515	6.04	0.000	1.037563	1.075006
gdpcapi	.8930956	.0232125	-4.35	0.000	.8487394	.93977
gdpcapj	1.145698	.0330602	4.71	0.000	1.0827	1.212363
op3	.9821558	.0291233	-0.61	0.544	.9267021	1.040928

Conditional (fixed-effects) logistic regression   Number of obs = 35204
Wald chi2(5) = 59.40
Prob > chi2 = 0.0000
Log pseudolikelihood = -13687.003   Pseudo R2 = 0.0327

(Std. Err. adjusted for clustering on countrypair)

gdppc	Odds Ratio	Robust Std. Err.	z	P>|z|	[95% Conf.	Interval]
popi	.9830191	.0056234	-2.99	0.003	.972059	.9941028
popj	1.058782	.0099109	6.10	0.000	1.039534	1.078386
gdpcapi	.8790629	.0241712	-4.69	0.000	.8329422	.9277373
gdpcapj	1.124352	.0322865	4.08	0.000	1.062819	1.189447
op4	2.902649	1.384093	2.23	0.025	1.140012	7.390601

Conditional (fixed-effects) logistic regression   Number of obs = 38388
Wald chi2(5) = 60.56
Prob > chi2 = 0.0000
Log pseudolikelihood = -14958.517   Pseudo R2 = 0.0304

(Std. Err. adjusted for clustering on countrypair)

gdppc	Odds Ratio	Robust Std. Err.	z	P>|z|	[95% Conf.	Interval]
popi	.9864805	.0055219	-2.43	0.015	.975717	.9973627
popj	1.059484	.0104258	5.87	0.000	1.039245	1.080116
gdpcapi	.8983147	.0225149	-4.28	0.000	.8552526	.943545
gdpcapj	1.146194	.032144	4.87	0.000	1.084893	1.210958
op5	.9468201	.0253272	-2.04	0.041	.8984585	.9977849

Conditional (fixed-effects) logistic regression   Number of obs = 38388
Wald chi2(5) = 55.23
Prob > chi2 = 0.0000
Log pseudolikelihood = -14966.424   Pseudo R2 = 0.0299

(Std. Err. adjusted for clustering on countrypair)

gdppc	Odds Ratio	Robust Std. Err.	z	P>|z|	[95% Conf.	Interval]
popi	.985394	.0055801	-2.60	0.009	.9745178	.9963917
popj	1.060236	.0105917	5.86	0.000	1.039679	1.0812
gdpcapi	.8911242	.0226073	-4.54	0.000	.8478984	.9365537
gdpcapj	1.152281	.0334901	4.88	0.000	1.088476	1.219826
op6	1.024584	.0147582	1.69	0.092	.9960628	1.053922

```
Conditional (fixed-effects) logistic regression   Number of obs   =      38388
                                                  Wald chi2(5)    =      62.28
                                                  Prob > chi2     =     0.0000
Log pseudolikelihood = -14900.847                 Pseudo R2       =     0.0341

                            (Std. Err. adjusted for clustering on countrypair)
```

gdppc	Odds Ratio	Robust Std. Err.	z	P>|z|	[95% Conf.	Interval]
popi	.9850459	.0055629	-2.67	0.008	.9742031	.9960095
popj	1.060974	.010739	5.85	0.000	1.040133	1.082232
gdpcapi	.8827524	.0225939	-4.87	0.000	.8395616	.9281652
gdpcapj	1.164355	.0333571	5.31	0.000	1.100778	1.231604
op7	1.046708	.0171913	2.78	0.005	1.01355	1.080951

```
Conditional (fixed-effects) logistic regression   Number of obs   =      38388
                                                  Wald chi2(5)    =      60.97
                                                  Prob > chi2     =     0.0000
Log pseudolikelihood = -14917.591                 Pseudo R2       =     0.0330

                            (Std. Err. adjusted for clustering on countrypair)
```

gdppc	Odds Ratio	Robust Std. Err.	z	P>|z|	[95% Conf.	Interval]
popi	.984942	.0055664	-2.68	0.007	.9740922	.9959126
popj	1.060685	.0106673	5.86	0.000	1.039982	1.0818
gdpcapi	.8853103	.0227641	-4.74	0.000	.8417991	.9310706
gdpcapj	1.159347	.0335502	5.11	0.000	1.09542	1.227004
op8	1.159254	.051695	3.31	0.001	1.062235	1.265133

```
Conditional (fixed-effects) logistic regression   Number of obs   =      37961
                                                  Wald chi2(5)    =      56.72
                                                  Prob > chi2     =     0.0000
Log pseudolikelihood = -14805.088                 Pseudo R2       =     0.0292

                            (Std. Err. adjusted for clustering on countrypair)
```

gdppc	Odds Ratio	Robust Std. Err.	z	P>|z|	[95% Conf.	Interval]
popi	.986313	.0055623	-2.44	0.015	.9754711	.9972753
popj	1.058407	.0100356	5.99	0.000	1.038919	1.07826
gdpcapi	.8942086	.0223403	-4.48	0.000	.8514771	.9390845
gdpcapj	1.142332	.0316906	4.80	0.000	1.081878	1.206164
op9	.9784782	.0272181	-0.78	0.434	.9265598	1.033306

Model – 4A

Conditional (fixed-effects) logistic regression Number of obs = 35564
Wald chi2(14) = 74.45
Prob > chi2 = 0.0000
Log pseudolikelihood = -13747.904 Pseudo R2 = 0.0376

(Std. Err. adjusted for clustering on countrypair)

gdppc	Odds Ratio	Robust Std. Err.	z	P>|z|	[95% Conf.	Interval]
popi	.9856913	.0056124	-2.53	0.011	.9747523	.996753
popj	1.059549	.0103332	5.93	0.000	1.039489	1.079997
gdpcapi	.8863752	.0242516	-4.41	0.000	.8400949	.9352051
gdpcapj	1.135106	.0347337	4.14	0.000	1.06903	1.205266
up0	1.073886	.0367397	2.08	0.037	1.004238	1.148363
up1	1.030595	.1055594	0.29	0.769	.8431464	1.259717
up2	.9851659	.0564616	-0.26	0.794	.8804922	1.102283
up3	.960096	.0236402	-1.65	0.098	.9148623	1.007566
up4	1.317129	.4062586	0.89	0.372	.7195838	2.410877
up5	.9512776	.0215565	-2.20	0.028	.9099522	.9944798
up6	.9826618	.0167923	-1.02	0.306	.9502946	1.016131
up7	1.035783	.0153299	2.38	0.018	1.006168	1.066269
up8	1.074695	.036005	2.15	0.032	1.006394	1.147632
up9	.9820373	.019604	-0.91	0.364	.9443562	1.021222

Variables:

up0: the average trade volume of *'Food and live animals'* industry from country *j* to country *i* divided by the GDP of country *j*
up1: the average trade volume of *'Beverages and tobacco'* industry from country *j* to country *i* divided by the GDP of country *j*
up2: the average trade volume of *'Crude materials, inedible, except fuels'* industry from country *j* to country *i* divided by the GDP of country *j*
up3: the average trade volume of *'Mineral fuels, lubricants and related materials'* industry from country *j* to country *i* divided by the GDP of country *j*
up4: the average trade volume of *'Animal and vegetable oils, fats and waxes'* industry from country *j* to country *i* divided by the GDP of country *j*
up5: the average trade volume of *'Chemicals and related products, n.e.s.'* industry from country *j* to country *i* divided by the GDP of country *j*
up6: the average trade volume of *'Manufactured goods classified chiefly by material'* industry from country *j* to country *i* divided by the GDP of country *j*
up7: the average trade volume of *'Machinery and transport equipment'* industry from country *j* to country *i* divided by the GDP of country *j*
up8: the average trade volume of *'Miscellaneous manufactured articles'* industry from country *j* to country *i* divided by the GDP of country *j*
up9: the average trade volume of *'Commodities and transactions not classified elsewhere in the SITC'* industry from country *j* to country *i* divided by the GDP of country *j*

```
Conditional (fixed-effects) logistic regression   Number of obs   =      35781
                                                  Wald chi2(14)   =      87.07
                                                  Prob > chi2     =     0.0000
Log pseudolikelihood = -13738.311                 Pseudo R2       =     0.0445

                              (Std. Err. adjusted for clustering on countrypair)
```

gdppc	Odds Ratio	Robust Std. Err.	z	P>|z|	[95% Conf.	Interval]
popi	.9869661	.0056364	-2.30	0.022	.9759805	.9980753
popj	1.05597	.0093658	6.14	0.000	1.037772	1.074487
gdpcapi	.8722154	.0244975	-4.87	0.000	.8254989	.9215757
gdpcapj	1.174567	.0371524	5.09	0.000	1.103961	1.249689
down0	1.085316	.0785232	1.13	0.258	.9418269	1.250665
down1	.7860394	.1595319	-1.19	0.236	.528065	1.170042
down2	.8719528	.0776846	-1.54	0.124	.7322462	1.038314
down3	.9916915	.0224786	-0.37	0.713	.9485986	1.036742
down4	2.525413	1.19761	1.95	0.051	.9969489	6.397232
down5	.8888145	.0369674	-2.83	0.005	.8192342	.9643045
down6	1.000327	.0224363	0.01	0.988	.9573055	1.045282
down7	1.052102	.0202159	2.64	0.008	1.013216	1.09248
down8	1.056352	.0689629	0.84	0.401	.9294773	1.200545
down9	.9964975	.0155259	-0.23	0.822	.9665272	1.027397

Variables:

down0: the average trade volume of *'Food and live animals'* industry from country *i* to country *j* divided by the GDP of country *j*

down1: the average trade volume of *'Beverages and tobacco'* industry from country *i* to country *j* divided by the GDP of country *j*

down2: the average trade volume of *'Crude materials, inedible, except fuels'* industry from country *i* to country *j* divided by the GDP of country *j*

down3: the average trade volume of *'Mineral fuels, lubricants and related materials'* industry from country *i* to country *j* divided by the GDP of country *j*

down4: the average trade volume of *'Animal and vegetable oils, fats and waxes'* industry from country *i* to country *j* divided by the GDP of country *j*

down5: the average trade volume of *'Chemicals and related products, n.e.s.'* industry from country *i* to country *j* divided by the GDP of country *j*

down6: the average trade volume of *'Manufactured goods classified chiefly by material'* industry from country *i* to country *j* divided by the GDP of country *j*

down7: the average trade volume of *'Machinery and transport equipment'* industry from country *i* to country *j* divided by the GDP of country *j*

down8: the average trade volume of *'Miscellaneous manufactured articles'* industry from country *i* to country *j* divided by the GDP of country *j*

down9: the average trade volume of *'Commodities and transactions not classified elsewhere in the SITC'* industry from country *i* to country *j* divided by the GDP of country *j*

Model – 4B

```
Conditional (fixed-effects) logistic regression   Number of obs   =      38388
                                                  Wald chi2(5)    =      59.69
                                                  Prob > chi2     =     0.0000
Log pseudolikelihood = -14941.317                 Pseudo R2       =     0.0315

                            (Std. Err. adjusted for clustering on countrypair)
```

gdppc	Odds Ratio	Robust Std. Err.	z	P>|z|	[95% Conf.	Interval]
popi	.9859078	.0055283	-2.53	0.011	.9751319	.9968028
popj	1.060567	.0106608	5.85	0.000	1.039876	1.081668
gdpcapi	.8917215	.0223666	-4.57	0.000	.8489439	.9366547
gdpcapj	1.143838	.0316455	4.86	0.000	1.083465	1.207574
up0	1.073003	.0318894	2.37	0.018	1.012287	1.137361

```
Conditional (fixed-effects) logistic regression   Number of obs   =      38192
                                                  Wald chi2(5)    =      55.04
                                                  Prob > chi2     =     0.0000
Log pseudolikelihood = -14901.542                 Pseudo R2       =     0.0296

                            (Std. Err. adjusted for clustering on countrypair)
```

gdppc	Odds Ratio	Robust Std. Err.	z	P>|z|	[95% Conf.	Interval]
popi	.9858536	.005551	-2.53	0.011	.9750337	.9967937
popj	1.060305	.0106086	5.85	0.000	1.039715	1.081302
gdpcapi	.8934555	.0223987	-4.49	0.000	.8506159	.9384526
gdpcapj	1.144344	.031732	4.86	0.000	1.08381	1.208259
up1	1.06526	.0930463	0.72	0.469	.8976484	1.264167

```
Conditional (fixed-effects) logistic regression   Number of obs   =      38388
                                                  Wald chi2(5)    =      55.10
                                                  Prob > chi2     =     0.0000
Log pseudolikelihood = -14975.988                 Pseudo R2       =     0.0293

                            (Std. Err. adjusted for clustering on countrypair)
```

gdppc	Odds Ratio	Robust Std. Err.	z	P>|z|	[95% Conf.	Interval]
popi	.9859001	.0056409	-2.48	0.013	.974906	.9970182
popj	1.060067	.0105538	5.86	0.000	1.039582	1.080955
gdpcapi	.8946512	.0223088	-4.46	0.000	.851978	.9394616
gdpcapj	1.143789	.0316891	4.85	0.000	1.083336	1.207616
up2	1.008123	.0548569	0.15	0.882	.9061408	1.121584

```
Conditional (fixed-effects) logistic regression   Number of obs   =      37329
                                                  Wald chi2(5)    =      57.12
                                                  Prob > chi2     =     0.0000
Log pseudolikelihood = -14570.644                 Pseudo R2       =     0.0294

                            (Std. Err. adjusted for clustering on countrypair)
```

gdppc	Odds Ratio	Robust Std. Err.	z	P>|z|	[95% Conf.	Interval]
popi	.9865566	.0055377	-2.41	0.016	.9757623	.9974703
popj	1.057754	.0100361	5.92	0.000	1.038266	1.077609
gdpcapi	.8936903	.0229082	-4.38	0.000	.8499004	.9397365
gdpcapj	1.144003	.0319664	4.81	0.000	1.083035	1.208404
up3	.9725303	.0257061	-1.05	0.292	.9234302	1.024241

```
Conditional (fixed-effects) logistic regression   Number of obs   =      36585
                                                  Wald chi2(5)    =      53.12
                                                  Prob > chi2     =     0.0000
Log pseudolikelihood = -14235.247                 Pseudo R2       =     0.0300

                           (Std. Err. adjusted for clustering on countrypair)
```

gdppc	Odds Ratio	Robust Std. Err.	z	P>|z|	[95% Conf. Interval]	
popi	.9835259	.0055841	-2.93	0.003	.9726419	.9945316
popj	1.060954	.0106469	5.90	0.000	1.04029	1.082028
gdpcapi	.8945889	.022936	-4.34	0.000	.8507459	.9406913
gdpcapj	1.121703	.0317395	4.06	0.000	1.061188	1.185668
up4	1.521745	.4572674	1.40	0.162	.844433	2.742322

```
Conditional (fixed-effects) logistic regression   Number of obs   =      38388
                                                  Wald chi2(5)    =      58.05
                                                  Prob > chi2     =     0.0000
Log pseudolikelihood = -14965.221                 Pseudo R2       =     0.0300

                           (Std. Err. adjusted for clustering on countrypair)
```

gdppc	Odds Ratio	Robust Std. Err.	z	P>|z|	[95% Conf. Interval]	
popi	.9861932	.005517	-2.49	0.013	.9754392	.9970658
popj	1.059736	.0104736	5.87	0.000	1.039406	1.080464
gdpcapi	.8964258	.0223799	-4.38	0.000	.8536179	.9413804
gdpcapj	1.14543	.0321147	4.84	0.000	1.084184	1.210135
up5	.9695977	.0160408	-1.87	0.062	.9386625	1.001552

```
Conditional (fixed-effects) logistic regression   Number of obs   =      38388
                                                  Wald chi2(5)    =      55.15
                                                  Prob > chi2     =     0.0000
Log pseudolikelihood = -14974.245                 Pseudo R2       =     0.0294

                           (Std. Err. adjusted for clustering on countrypair)
```

gdppc	Odds Ratio	Robust Std. Err.	z	P>|z|	[95% Conf. Interval]	
popi	.9857588	.0055662	-2.54	0.011	.9749093	.9967289
popj	1.060166	.0105762	5.86	0.000	1.039638	1.081099
gdpcapi	.8932035	.0225838	-4.47	0.000	.8500189	.938582
gdpcapj	1.147196	.0330537	4.77	0.000	1.084207	1.213844
up6	1.008651	.0116459	0.75	0.456	.9860815	1.031736

```
Conditional (fixed-effects) logistic regression   Number of obs   =      38388
                                                  Wald chi2(5)    =      57.50
                                                  Prob > chi2     =     0.0000
Log pseudolikelihood = -14943.429                 Pseudo R2       =     0.0314

                           (Std. Err. adjusted for clustering on countrypair)
```

gdppc	Odds Ratio	Robust Std. Err.	z	P>|z|	[95% Conf. Interval]	
popi	.9855255	.0055506	-2.59	0.010	.9747064	.9964647
popj	1.06088	.010731	5.84	0.000	1.040055	1.082122
gdpcapi	.8882761	.0224461	-4.69	0.000	.8453542	.9333772
gdpcapj	1.151536	.0318629	5.10	0.000	1.090749	1.21571
up7	1.028403	.0147894	1.95	0.051	.9998211	1.057802

Conditional (fixed-effects) logistic regression   Number of obs = 38388
Wald chi2(5) = 54.67
Prob > chi2 = 0.0000
Log pseudolikelihood = -14964.693   Pseudo R2 = 0.0300

(Std. Err. adjusted for clustering on countrypair)

gdppc	Odds Ratio	Robust Std. Err.	z	P>|z|	[95% Conf.	Interval]
popi	.9857523	.0055494	-2.55	0.011	.9749355	.9966892
popj	1.060213	.0105814	5.86	0.000	1.039676	1.081157
gdpcapi	.8926549	.0226592	-4.47	0.000	.8493304	.9381894
gdpcapj	1.146958	.0328369	4.79	0.000	1.084371	1.213157
up8	1.04944	.0451683	1.12	0.262	.9645426	1.141809

Conditional (fixed-effects) logistic regression   Number of obs = 38143
Wald chi2(5) = 55.24
Prob > chi2 = 0.0000
Log pseudolikelihood = -14867.108   Pseudo R2 = 0.0295

(Std. Err. adjusted for clustering on countrypair)

gdppc	Odds Ratio	Robust Std. Err.	z	P>|z|	[95% Conf.	Interval]
popi	.9863653	.0055791	-2.43	0.015	.9754908	.997361
popj	1.060127	.010559	5.86	0.000	1.039633	1.081026
gdpcapi	.8949431	.0223176	-4.45	0.000	.8522531	.9397715
gdpcapj	1.140714	.0314709	4.77	0.000	1.08067	1.204093
up9	.9865241	.0170939	-0.78	0.434	.9535832	1.020603

Conditional (fixed-effects) logistic regression   Number of obs = 38346
Wald chi2(5) = 56.35
Prob > chi2 = 0.0000
Log pseudolikelihood = -14935.166   Pseudo R2 = 0.0302

(Std. Err. adjusted for clustering on countrypair)

gdppc	Odds Ratio	Robust Std. Err.	z	P>|z|	[95% Conf.	Interval]
popi	.9859122	.0055461	-2.52	0.012	.9751018	.9968425
popj	1.060532	.0106301	5.86	0.000	1.039901	1.081572
gdpcapi	.8904293	.0224109	-4.61	0.000	.8475705	.9354553
gdpcapj	1.143301	.0316297	4.84	0.000	1.082959	1.207006
down0	1.063278	.0433756	1.50	0.133	.9815734	1.151784

Conditional (fixed-effects) logistic regression   Number of obs = 38237
Wald chi2(5) = 56.38
Prob > chi2 = 0.0000
Log pseudolikelihood = -14890.777   Pseudo R2 = 0.0294

(Std. Err. adjusted for clustering on countrypair)

gdppc	Odds Ratio	Robust Std. Err.	z	P>|z|	[95% Conf.	Interval]
popi	.985791	.0055556	-2.54	0.011	.9749621	.9967401
popj	1.058687	.0101475	5.95	0.000	1.038983	1.078763
gdpcapi	.8892639	.022586	-4.62	0.000	.8460798	.934652
gdpcapj	1.150382	.0329173	4.90	0.000	1.087641	1.216743
down1	1.14929	.1102872	1.45	0.147	.9522417	1.387113

```
Conditional (fixed-effects) logistic regression   Number of obs   =      38346
                                                  Wald chi2(5)    =      55.66
                                                  Prob > chi2     =     0.0000
Log pseudolikelihood =  -14963.18                 Pseudo R2       =     0.0295

                            (Std. Err. adjusted for clustering on countrypair)
```

gdppc	Odds Ratio	Robust Std. Err.	z	P>|z|	[95% Conf. Interval]	
popi	.9860955	.0055325	-2.50	0.013	.9753114	.9969988
popj	1.059905	.0105042	5.87	0.000	1.039516	1.080694
gdpcapi	.8959104	.022298	-4.42	0.000	.853256	.940697
gdpcapj	1.142803	.031729	4.81	0.000	1.082277	1.206714
down2	.9489004	.0612632	-0.81	0.417	.8361133	1.076902

```
Conditional (fixed-effects) logistic regression   Number of obs   =      37502
                                                  Wald chi2(5)    =      55.99
                                                  Prob > chi2     =     0.0000
Log pseudolikelihood = -14598.756                 Pseudo R2       =     0.0289

                            (Std. Err. adjusted for clustering on countrypair)
```

gdppc	Odds Ratio	Robust Std. Err.	z	P>|z|	[95% Conf. Interval]	
popi	.9869702	.0055979	-2.31	0.021	.9760592	.9980032
popj	1.057503	.0098685	5.99	0.000	1.038337	1.077023
gdpcapi	.894909	.0226148	-4.39	0.000	.8516645	.9403493
gdpcapj	1.145155	.0327264	4.74	0.000	1.082776	1.211128
down3	.9963285	.0176117	-0.21	0.835	.9624012	1.031452

```
Conditional (fixed-effects) logistic regression   Number of obs   =      36555
                                                  Wald chi2(5)    =      61.37
                                                  Prob > chi2     =     0.0000
Log pseudolikelihood = -14264.069                 Pseudo R2       =     0.0327

                            (Std. Err. adjusted for clustering on countrypair)
```

gdppc	Odds Ratio	Robust Std. Err.	z	P>|z|	[95% Conf. Interval]	
popi	.985764	.0055802	-2.53	0.011	.9748873	.9967619
popj	1.057747	.009804	6.06	0.000	1.038705	1.077138
gdpcapi	.8778869	.0235494	-4.86	0.000	.8329233	.9252777
gdpcapj	1.143062	.0322054	4.75	0.000	1.081651	1.207958
down4	2.541145	1.26066	1.88	0.060	.9610571	6.719078

```
Conditional (fixed-effects) logistic regression   Number of obs   =      38388
                                                  Wald chi2(5)    =      58.61
                                                  Prob > chi2     =     0.0000
Log pseudolikelihood = -14963.924                 Pseudo R2       =     0.0300

                            (Std. Err. adjusted for clustering on countrypair)
```

gdppc	Odds Ratio	Robust Std. Err.	z	P>|z|	[95% Conf. Interval]	
popi	.9864712	.0055359	-2.43	0.015	.9756805	.9973814
popj	1.059595	.0104611	5.86	0.000	1.039289	1.080299
gdpcapi	.8979779	.0225414	-4.29	0.000	.8548668	.9432632
gdpcapj	1.145393	.0318414	4.88	0.000	1.084655	1.209532
down5	.9578697	.029274	-1.41	0.159	.9021782	1.016999

Conditional (fixed-effects) logistic regression   Number of obs = 38388
Wald chi2(5) = 56.28
Prob > chi2 = 0.0000
Log pseudolikelihood = -14953.633   Pseudo R2 = 0.0307

(Std. Err. adjusted for clustering on countrypair)

gdppc	Odds Ratio	Robust Std. Err.	z	P>|z|	[95% Conf.	Interval]
popi	.9851522	.0055659	-2.65	0.008	.9743034	.9961218
popj	1.060115	.0105653	5.86	0.000	1.039608	1.081026
gdpcapi	.8903874	.0226045	-4.57	0.000	.8471675	.9358122
gdpcapj	1.154903	.033531	4.96	0.000	1.091018	1.222528
down6	1.040464	.0230842	1.79	0.074	.9961896	1.086706

Conditional (fixed-effects) logistic regression   Number of obs = 38388
Wald chi2(5) = 62.20
Prob > chi2 = 0.0000
Log pseudolikelihood = -14885.912   Pseudo R2 = 0.0351

(Std. Err. adjusted for clustering on countrypair)

gdppc	Odds Ratio	Robust Std. Err.	z	P>|z|	[95% Conf.	Interval]
popi	.9850042	.0055561	-2.68	0.007	.9741744	.9959545
popj	1.06053	.0106333	5.86	0.000	1.039893	1.081577
gdpcapi	.8826803	.0225828	-4.88	0.000	.8395103	.9280701
gdpcapj	1.168846	.0338437	5.39	0.000	1.104361	1.237097
down7	1.044657	.0207124	2.20	0.028	1.00484	1.086052

Conditional (fixed-effects) logistic regression   Number of obs = 38388
Wald chi2(5) = 60.20
Prob > chi2 = 0.0000
Log pseudolikelihood = -14920.833   Pseudo R2 = 0.0328

(Std. Err. adjusted for clustering on countrypair)

gdppc	Odds Ratio	Robust Std. Err.	z	P>|z|	[95% Conf.	Interval]
popi	.9850689	.0055382	-2.68	0.007	.9742738	.9959835
popj	1.060563	.0106404	5.86	0.000	1.039912	1.081624
gdpcapi	.8860625	.0225726	-4.75	0.000	.8429074	.9314272
gdpcapj	1.158869	.0331514	5.15	0.000	1.095682	1.225701
down8	1.10581	.0584893	1.90	0.057	.9969147	1.226599

Conditional (fixed-effects) logistic regression   Number of obs = 38125
Wald chi2(5) = 56.90
Prob > chi2 = 0.0000
Log pseudolikelihood = -14881.213   Pseudo R2 = 0.0291

(Std. Err. adjusted for clustering on countrypair)

gdppc	Odds Ratio	Robust Std. Err.	z	P>|z|	[95% Conf.	Interval]
popi	.9860462	.0055251	-2.51	0.012	.9752764	.9969349
popj	1.058399	.0100289	5.99	0.000	1.038924	1.078239
gdpcapi	.8929571	.0223042	-4.53	0.000	.8502945	.9377602
gdpcapj	1.14542	.0318349	4.89	0.000	1.084693	1.209546
down9	.9894015	.02401	-0.44	0.661	.9434443	1.037597

Model – 5A

Conditional (fixed-effects) logistic regression

Number of obs = 34152
Wald chi2(24) = 103.68
Prob > chi2 = 0.0000
Log pseudolikelihood = -13003.79
Pseudo R2 = 0.0508

(Std. Err. adjusted for clustering on countrypair)

gdppc	Odds Ratio	Robust Std. Err.	z	P>|z|	[95% Conf.	Interval]
popi	.9862904	.0057434	-2.37	0.018	.9750975	.9976118
popj	1.057007	.0095861	6.11	0.000	1.038384	1.075963
gdpcapi	.8716151	.0258194	-4.64	0.000	.822451	.9237181
gdpcapj	1.153894	.0381257	4.33	0.000	1.081537	1.231092
down0	1.115541	.0801347	1.52	0.128	.9690356	1.284197
down1	.7752998	.1380634	-1.43	0.153	.5468773	1.099131
down2	.8568466	.0696757	-1.90	0.057	.7306113	1.004893
down3	.987575	.0238001	-0.52	0.604	.9420123	1.035342
down4	2.66333	1.301785	2.00	0.045	1.021819	6.941862
down5	.9010023	.03897	-2.41	0.016	.8277704	.9807129
down6	.9897329	.0297168	-0.34	0.731	.9331697	1.049725
down7	1.046509	.0228644	2.08	0.037	1.002641	1.092296
down8	1.051929	.0623129	0.85	0.393	.936621	1.181432
down9	1.001987	.0132906	0.15	0.881	.9762739	1.028378
up0	1.069324	.0356193	2.01	0.044	1.001742	1.141466
up1	1.012197	.1159974	0.11	0.916	.8085702	1.267105
up2	.983808	.0532663	-0.30	0.763	.8847565	1.093949
up3	.9500155	.0242293	-2.01	0.044	.9036943	.9987111
up4	1.353834	.4159094	0.99	0.324	.741428	2.472076
up5	.9663035	.0214029	-1.55	0.122	.925252	1.009176
up6	.9829626	.026643	-0.63	0.526	.932106	1.036594
up7	1.014763	.0142438	1.04	0.296	.9872259	1.043067
up8	1.096142	.0480623	2.09	0.036	1.005876	1.194508
up9	.98262	.0197779	-0.87	0.384	.9446107	1.022159

Model – 5B

Conditional (fixed-effects) logistic regression

Number of obs = 38346
Wald chi2(6) = 60.56
Prob > chi2 = 0.0000
Log pseudolikelihood = -14909.425
Pseudo R2 = 0.0319

(Std. Err. adjusted for clustering on countrypair)

gdppc	Odds Ratio	Robust Std. Err.	z	P>|z|	[95% Conf.	Interval]
popi	.9858709	.0055403	-2.53	0.011	.9750717	.9967896
popj	1.060755	.0106786	5.86	0.000	1.04003	1.081892
gdpcapi	.8890007	.0224794	-4.65	0.000	.846016	.9341695
gdpcapj	1.14387	.031599	4.87	0.000	1.083584	1.20751
up0	1.063658	.0308427	2.13	0.033	1.004893	1.12586
down0	1.038682	.0419437	0.94	0.347	.9596426	1.12423

Conditional (fixed-effects) logistic regression

Number of obs = 38041
Wald chi2(6) = 56.37
Prob > chi2 = 0.0000
Log pseudolikelihood = -14816.998
Pseudo R2 = 0.0297

(Std. Err. adjusted for clustering on countrypair)

gdppc	Odds Ratio	Robust Std. Err.	z	P>|z|	[95% Conf.	Interval]
popi	.9856982	.0055737	-2.55	0.011	.9748342	.9966832
popj	1.058883	.0101931	5.94	0.000	1.039092	1.079051
gdpcapi	.888271	.0226634	-4.64	0.000	.8449439	.9338198
gdpcapj	1.150865	.0330227	4.90	0.000	1.087928	1.217442
up1	1.033896	.0978178	0.35	0.725	.8589029	1.244543
down1	1.142897	.1137813	1.34	0.180	.9402978	1.389148

```
Conditional (fixed-effects) logistic regression   Number of obs   =      38346
                                                  Wald chi2(6)    =      55.60
                                                  Prob > chi2     =     0.0000
Log pseudolikelihood = -14962.742                 Pseudo R2       =     0.0295

                            (Std. Err. adjusted for clustering on countrypair)
```

gdppc	Odds Ratio	Robust Std. Err.	z	P>|z|	[95% Conf.	Interval]
popi	.985919	.0056483	-2.48	0.013	.9749105	.9970519
popj	1.059928	.010508	5.87	0.000	1.039532	1.080725
gdpcapi	.8957262	.0223234	-4.42	0.000	.8530245	.9405655
gdpcapj	1.142967	.0317759	4.81	0.000	1.082354	1.206974
up2	1.015512	.0544181	0.29	0.774	.9142644	1.127972
down2	.9461335	.0606599	-0.86	0.388	.834409	1.072818

```
Conditional (fixed-effects) logistic regression   Number of obs   =      36610
                                                  Wald chi2(6)    =      57.85
                                                  Prob > chi2     =     0.0000
Log pseudolikelihood = -14249.597                 Pseudo R2       =     0.0294

                            (Std. Err. adjusted for clustering on countrypair)
```

gdppc	Odds Ratio	Robust Std. Err.	z	P>|z|	[95% Conf.	Interval]
popi	.9874308	.0056103	-2.23	0.026	.9764958	.9984882
popj	1.056068	.0095501	6.03	0.000	1.037515	1.074953
gdpcapi	.8927966	.0232083	-4.36	0.000	.8484485	.9394626
gdpcapj	1.146728	.0331554	4.74	0.000	1.083551	1.213588
up3	.9720182	.0253684	-1.09	0.277	.9235474	1.023033
down3	1.000253	.0186385	0.01	0.989	.9643815	1.037459

```
Conditional (fixed-effects) logistic regression   Number of obs   =      35204
                                                  Wald chi2(6)    =      59.46
                                                  Prob > chi2     =     0.0000
Log pseudolikelihood = -13681.847                 Pseudo R2       =     0.0331

                            (Std. Err. adjusted for clustering on countrypair)
```

gdppc	Odds Ratio	Robust Std. Err.	z	P>|z|	[95% Conf.	Interval]
popi	.9834117	.0055856	-2.95	0.003	.9725249	.9944203
popj	1.058787	.0099507	6.08	0.000	1.039462	1.07847
gdpcapi	.8789243	.0241244	-4.70	0.000	.8328906	.9275023
gdpcapj	1.123957	.0322182	4.08	0.000	1.062552	1.188911
up4	1.458502	.4318344	1.27	0.202	.8163619	2.605742
down4	2.276048	1.127661	1.66	0.097	.8618988	6.010445

```
Conditional (fixed-effects) logistic regression   Number of obs   =      38388
                                                  Wald chi2(6)    =      61.25
                                                  Prob > chi2     =     0.0000
Log pseudolikelihood = -14958.114                 Pseudo R2       =     0.0304

                            (Std. Err. adjusted for clustering on countrypair)
```

gdppc	Odds Ratio	Robust Std. Err.	z	P>|z|	[95% Conf.	Interval]
popi	.9865281	.0055303	-2.42	0.016	.9757483	.997427
popj	1.05946	.0104241	5.87	0.000	1.039225	1.080089
gdpcapi	.89857	.0225967	-4.25	0.000	.8553552	.9439683
gdpcapj	1.146217	.0321012	4.87	0.000	1.084995	1.210893
up5	.9777312	.0137247	-1.60	0.109	.9511979	1.005005
down5	.9661533	.0305523	-1.09	0.276	.9080899	1.027929

```
Conditional (fixed-effects) logistic regression   Number of obs   =      38388
                                                  Wald chi2(6)    =      56.79
                                                  Prob > chi2     =     0.0000
Log pseudolikelihood = -14949.264                 Pseudo R2       =     0.0310

                            (Std. Err. adjusted for clustering on countrypair)
```

gdppc	Odds Ratio	Robust Std. Err.	z	P>|z|	[95% Conf.	Interval]
popi	.9853156	.0055463	-2.63	0.009	.9745049	.9962463
popj	1.059912	.0105252	5.86	0.000	1.039482	1.080743
gdpcapi	.8920033	.0227868	-4.47	0.000	.8484416	.9378016
gdpcapj	1.151779	.0337643	4.82	0.000	1.087467	1.219894
up6	.9833471	.0209464	-0.79	0.430	.9431382	1.02527
down6	1.055995	.036825	1.56	0.118	.9862303	1.130694

Conditional (fixed-effects) logistic regression   Number of obs = 38388
Wald chi2(6) = 62.81
Prob > chi2 = 0.0000
Log pseudolikelihood = -14885.776   Pseudo R2 = 0.0351

(Std. Err. adjusted for clustering on countrypair)

gdppc	Odds Ratio	Robust Std. Err.	z	P>|z|	[95% Conf.	Interval]
popi	.9849946	.0055574	-2.68	0.007	.9741623	.9959474
popj	1.06058	.010646	5.86	0.000	1.039919	1.081653
gdpcapi	.8825071	.0226165	-4.88	0.000	.8392745	.9279668
gdpcapj	1.168773	.0338189	5.39	0.000	1.104334	1.236972
up7	1.002105	.014753	0.14	0.886	.9736027	1.031441
down7	1.043376	.0249593	1.78	0.076	.9955862	1.093461

Conditional (fixed-effects) logistic regression   Number of obs = 38388
Wald chi2(6) = 62.73
Prob > chi2 = 0.0000
Log pseudolikelihood = -14909.547   Pseudo R2 = 0.0336

(Std. Err. adjusted for clustering on countrypair)

gdppc	Odds Ratio	Robust Std. Err.	z	P>|z|	[95% Conf.	Interval]
popi	.9848235	.0055605	-2.71	0.007	.9739852	.9957823
popj	1.06071	.0106691	5.86	0.000	1.040004	1.081828
gdpcapi	.8842375	.0227185	-4.79	0.000	.8408126	.9299051
gdpcapj	1.161698	.0336262	5.18	0.000	1.097627	1.22951
up8	1.0462	.0394512	1.20	0.231	.9716655	1.126452
down8	1.106246	.0491596	2.27	0.023	1.013972	1.206917

Conditional (fixed-effects) logistic regression   Number of obs = 37961
Wald chi2(6) = 56.77
Prob > chi2 = 0.0000
Log pseudolikelihood = -14804.937   Pseudo R2 = 0.0292

(Std. Err. adjusted for clustering on countrypair)

gdppc	Odds Ratio	Robust Std. Err.	z	P>|z|	[95% Conf.	Interval]
popi	.9863684	.0055803	-2.43	0.015	.9754916	.9973664
popj	1.058403	.0100374	5.99	0.000	1.038911	1.07826
gdpcapi	.8941807	.0223419	-4.48	0.000	.8514464	.9390599
gdpcapj	1.142166	.0316591	4.80	0.000	1.081771	1.205933
up9	.9873964	.0171348	-0.73	0.465	.9543775	1.021558
down9	.9933998	.0222507	-0.30	0.767	.9507326	1.037982
