# Supplementary figures and images for: Shock propagation channels behind the global economic contagion network. The role of economic sectors and the direction of trade
Source: PLoS One. 2021 Oct 20;16(10):e0258309. doi: 10.1371/journal.pone.0258309 (PMC8528308; doi:10.1371/journal.pone.0258309)

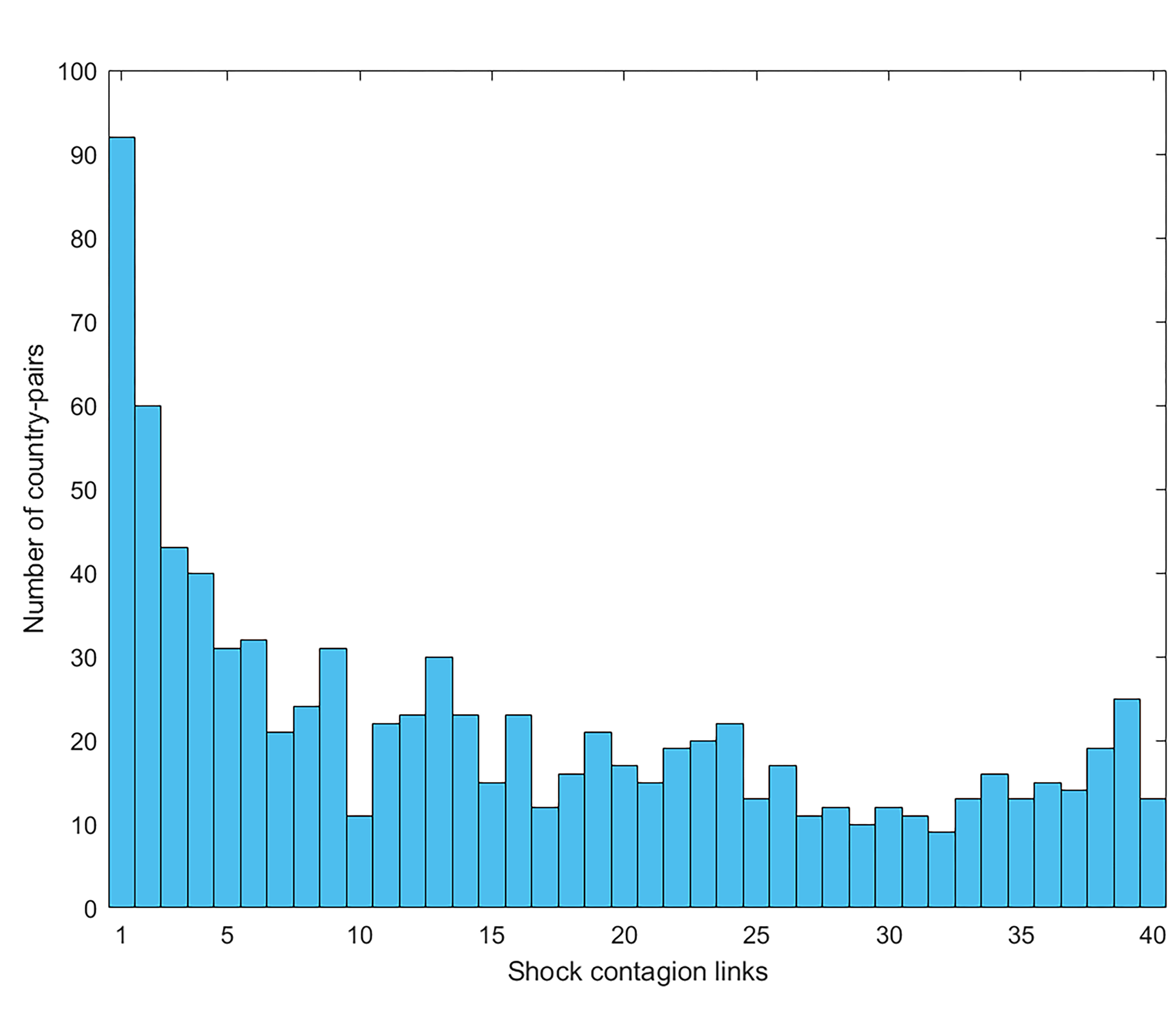

Supplement: S1 Fig — The figure is a histogram describing the distribution of observed shock-transmission events within country-pairs. For all (directed) country pairs we have 42 observations (time periods). The horizontal axis measures the number of time periods (from 1 to 41) and the bars at every value represent the number of country-pairs for which shock-transmission is observed exactly that many times as the number of time periods on the horizontal axis. We excluded those country-pairs from the figure for which we either do not observe shock transmission at all (at 0) or we observe transmission in every period (at 42), as these country-pairs are also dropped from the conditional logit regressions. (TIF) [file pone.0258309.s004.tif]
