# Supplementary material for: Shock propagation channels behind the global economic contagion network. The role of economic sectors and the direction of trade
Source: PLoS One. 2021 Oct 20;16(10):e0258309. doi: 10.1371/journal.pone.0258309 (PMC8528308; doi:10.1371/journal.pone.0258309)
Supplement: Attachment — Submitted filename: Response to Reviewers.pdf [file pone.0258309.s006.pdf]

Response to Reviewers

First, we would like to thank for the further valuable comments of the reviewer. We considered these comments and reviewed the manuscript accordingly, hoping that the concerns of Reviewer#1 have been addressed. We identified three layers in which our paper can address the issues raised by the reviewer and refer to these below, also pointing to the respective changes made to the manuscript. The reviewer comment is typed in italic while our answers are in normal font.

Reviewer #1: The authors respond my issues, though some slight concerns remaining. The aspects about causality and effect of regression (logic regression has limitation in high-dimensional data) remains unclear to this reviewer. In light of the spirit of PLOS ONE, this manuscript has some interesting point and can be publishable after improvement on above two aspects.

We identify a specific, primarily technical layer mentioned in the parentheses of the reviewer comment: this refers to the limitations of using logistic regression with high-dimensional data. With respect to this issue, we would like to note that high-dimensionality does not arise in our estimations, as the number of variables included in any of the regressions is magnitudes lower than the number of observations. We pointed to this in the manuscript, by inserting the following paragraph (~line 210).

‘Recent studies also call attention on the limitations of applying logistic regression in the case of high-dimensional data [59]. However, this limitation is not binding in this study, since the regression analysis employs 37 variables for approximately 38,000 observations, meaning that the condition for high-dimensionality does not apply. Moreover, these variables are not used simultaneously in the estimations (the most extensive estimation includes 24 variables and runs with 34,152 observations).’

As a second layer of the reviewer comment, we identified possible limitations with respect to potential endogeneity in the regressions, as the assumed effect (trade channels affect shock transmission) may be reversed (shocks affect trade patterns). We refer to this limitation in the discussion part, among other limitations of the study, by inserting the following paragraph (~756).

‘While the use of the fixed-effects panel model in this study reduces the risk of bias arising from omitted variables, it has limitations in handling potential endogeneity in the data. Our estimation procedure builds on the assumption that trade relationships are exogenous to shock transmission, so the latter do not affect the former, however, we can not rule this option out. This reverse causality would mean that shocks hitting the economies affect their economic processes, which in turn affect the trade patterns. While we can not rule this effect out, we argue that the assumption of trade links channelling shocks is stronger and are in line with economic models as already discussed under the upstream and downstream channels of shock propagation in the Results section. At the same time, the potential mechanisms behind the reverse causality are indirect and unfold over much longer periods of time.’

Finally, a third layer of the reviewer comment drove us towards potential controversies around the concept of Granger causality on which shock transmission is based in our paper. Although this is

a widely accepted tool in time series analysis, there are clear limitations which we mentioned in the manuscript when introducing the method, by inserting the following paragraph (~line 131).

‘While Granger causality tests are widely used in time series analysis as a method to identify casual relationships between observed variables, it is important to emphasise its limitations. Although Granger causality is suitable for detecting co-movement between time series and reflects the sequence of events, these identified cross-period co-movements does not always represent real causal relationships. The opposing views on the existence of actual causality and the different philosophical approaches to its identification are reviewed by e.g. [60, 61].’
