# Supplementary material for: Shock propagation channels behind the global economic contagion network. The role of economic sectors and the direction of trade
Source: PLoS One. 2021 Oct 20;16(10):e0258309. doi: 10.1371/journal.pone.0258309 (PMC8528308; doi:10.1371/journal.pone.0258309)
Supplement: Attachment — Submitted filename: ResponseToReviewers.docx [file pone.0258309.s005.docx]

**Answers to reviewer comments**

First of all, we would like to thank for all the valuable and insightful comments of the two reviewers. We have learned a lot from them, and reviewed the manuscript accordingly, hoping that it gained a lot in clarity and the interpretation of the results. In what follows, we reflect on the reviewers’ comments one-by-one and also point to the respective changes made to the manuscript. The reviewer comments are typed in italic while our answers are in normal font.

**Reviewer #1**

*- Network science suggests a new technical frontier to study economics, particularly international trade, and there are many existing papers looking into this direction. For contagion networks, in addition to the links as this paper paying most attention, network analysis of directed or bi-directed links could well tell a lot of stories behind.*

Thanks for this note. In order to emphasize this aspect, we inserted some references about network approaches in the analysis of shock contagion into the introduction (~ line 50).

*- The regression methods including logit regression, are rather standard. Therefore, these methods can only tell behavior over links, without a global comprehension as a network. To explore synchronization in a contagion network, the analytical tools presented in this paper are not sufficient.*

Indeed, the main methodological vehicle in this study is the logit regression which focuses on edge-level association between phenomena. In order to augment this focus, we inserted a topological analysis of the two networks under consideration: the shock-contagion network and the relative trade openness network. This analysis includes visualization as well as a comparison among the topological properties of these networks as much as possible (the content of the edge-weights establish some limitation to this comparison). We believe that this additional analysis enriches the descriptive part of the paper, makes the study more complete in terms of network analysis and also provides a good background for understanding the right-hand side and the left-hand side key variables in the regression models.

*- The openness in trade is equivalently measured by the incoming and outgoing ratio of a network node, which could not fully comprehend the behavior according to network science (or (Google) search in the network).*

The chosen trade openness measure (which we consistently relabeled as *relative trade openness* in the revised manuscript) is built on standard openness measures. Apart from distinguishing between upstream and downstream connections, this construction that we use implies a direction to trade relationships as it measures the relative importance of a given trade connection for the target country, where the latter is defined on the basis of the shock contagion network. This way, we rule out size effects from the estimations and put more weight to those connections which are intensive relative to the size of the shock-receiving country. We emphasized this approach and the rationale behind our choice in the section *Model variants: upstream and downstream channels, sectors*.

*- Due to reviewer's limited expertise in economics and trade, the contributions to economic behavior could not be well judged, though this reviewer could not identify new finding. In light of limited data analytical tools and no novel application of these tools in this paper, this reviewer suggests authors seek publication of this paper in the economy journals, rather than PLOS ONE.*

We emphasized both in the abstract and the conclusion that the main contribution of the paper is to reveal the finer details of shock propagation channels than just looking at aggregate bilateral trade, and point out those sectors and directions which makes it more likely for shocks to spread between countries. Also, we feel that the included topological analysis made the paper more relevant from the complex systems perspective as well.

**Reviewer #2**

*My main comment is related to clarifying the quantities and concepts that are being used by the authors. In particular, the authors introduce the concept of shock contagion, Granger causality, trade openness, synchronisation, upstream and downstream contagion, and substantially they evaluate two networks, one (static) extracted from GDP time series through Granger causality and one multi-layer networks (dynamic) related to trade between different countries in different sectors.*

*On this point, I think the authors should try to present an exploratory analysis of these two networks, describing their basic network properties. This would also allow to put the regression results in perspective.*

Thanks for this note and suggestion. Indeed, the analysis have exclusively focused on the logit regression and little has been exposed about the two networks that are in the background of the left and right hand sides of the regression equation. In order to fill this gap, we inserted a topological analysis of the two networks into the section of descriptive results. This analysis includes visualization as well as a comparison among the topological properties of these networks as much as possible (the content of the edge-weights establish some limitation to this comparison). We believe that this additional analysis enriches the descriptive part of the paper, makes the study more complete in terms of network analysis and also provides a good background for understanding the right-hand side and the left-hand side key variables in the regression models.

*Secondly, I think the authors should better clarify how the concepts they define are related to the two networks, e.g. shock contagion is derived from GDP time series but it is referred to also as synchronisation, then they use the term upstream and downstream shocks, referring to trade relationships, but it is not entirely clear how these labels are assigned to individual links. I believe the authors have tried to clarify these terms but I think the overall structure of the methodology could be improved.*

Thanks for this observation. We tried to render the exposition of the methods and indicators more concise by modifying the wording and adding clarifying sentences. Also, we made a consistent labeling of shock contagion network and links for the dependent variable and relative trade openness network and links for the key independent variables. Also, we clarified the Introduction in order to distinguish between synchronization, shock contagion and trade links.

*Further, if the authors introduced a more comprehensive general model which encompassed Model#A and Model#B the paper would probably be more accessible and intuitive, but this is a personal suggestion rather than a request for a revision. On this point the attempt to clarify the multiple tested models in Table 3 is partially successful.*

Thanks for this note. We would like to emphasize that models labelled with A are always more general than models labelled with B. In this sense, estimating a model with specifications #A and #B included would mean running model #A. Also, in the case of aggregate and sector level estimations #A and #B models do not refer to the same (or similar) specifications: for example, Model 2B includes only the key variable on the RHS plus the controls, while Model 2A adds opposite direction (upstream to downstream and vice versa). However, Model 3B includes only the bidirectional sector-level trade on the RHS (plus controls), while Model 3A adds all other sectors to the RHS as well, but in the same direction. We added a similar note to the discussion of Table 3 and referred to the regression tables to clarify the given specifications in the background.
